# Influence of Diet upon the Mother’s Milk

**Published:** 1869-01

**Authors:** 


### Selections.

Influence of Diet upon the Mother's Milk.—Dr. Subotin, of Petersburg (*Vierteljahrsshrift f. d. Prakt. Heilk.*, No. xxv, 1868), has instituted a series of experiments in regard to the influence of diet upon the quantity and quality of mother's milk, and his conclusions are as follows: 1st. That the daily yield of milk is increased by animal food, and is diminished by a diet of vegetables. A marked diminution of the milk, and, when persisted in, an entire suppression, is shown when food of a fatty nature has been given only. 2d. The relative proportions of the elements which compose the milk are influenced by the character of the food. The amount of solid matter is increased by an animal diet, and the fatty material is shown by this increase. The increase of casein is less marked. The increase of these substances in the milk is absolute, not relative; animal food increases the daily amount of the milk secretion. There is scarcely an appreciable change of the proportion of the albuminous and saline ingredients. Bensch supposed that the saccharine matter of the milk was reduced by the use of an animal diet, but it is found not to be so. The experiments of Drs. Bensch, Playfair, and others, that the fatty constituents of the milk are increased by vegetable food, and by an animal diet diminished, are not confirmed by him. The solid properties of the milk, especially the butyraceous, are but relatively increased, and at the same time a decrease in the sugar is shown. 3d. From these observations it would seem that the fatty matter of the milk is created, for the most part, from albumen.

